# Cost-effectiveness of Kang Ai injection plus chemotherapy vs. Shenqi Fuzheng injection plus chemotherapy in the first-line treatment of advanced non-small cell lung cancer

**DOI:** 10.3389/fmed.2024.1363484

**Published:** 2024-05-01

**Authors:** Junjie Zhu, Lei Tian

**Affiliations:** ^1^Department of Pharmacoeconomics, School of International Pharmaceutical Business, China Pharmaceutical University, Nanjing, China; ^2^Center for Pharmacoeconomics and Outcomes Research, China Pharmaceutical University, Nanjing, China

**Keywords:** advanced non-small cell lung cancer, Kang Ai injection, Shenqi Fuzheng injection, Markov model, cost-effectiveness

## Abstract

**Objective:**

This study aimed to evaluate the cost-effectiveness of two Chinese patent medicines, including Kang Ai injection and Shenqi Fuzheng injection with each combined with platinum-based chemotherapy as the first-line treatment for patients with advanced non-small cell lung cancer (NSCLC) in China.

**Methods:**

From Chinese healthcare system perspective, a three state Markov model with a cycle of 3 weeks and a 10-year horizon was constructed to derive the incremental cost-effectiveness ratio (ICER). Since only individual patient data of progression-free survival (PFS) of Kang Ai injection group can be obtained, we extrapolated median overall survival (mOS) of Kang Ai injection group and median progression-free survival (mPFS) and mOS of Shenqi Fuzheng injection group based on published literature and methods. Then survival curves were estimated by the method of declining exponential approximation of life expectancy (DEALE), which is based on the assumption that survival follows a declining exponential function. We performed one-way sensitivity analysis and probabilistic sensitivity analysis to test the robustness. Additionally, a scenario analysis was adopted to investigate the impact of using best-fitting distribution for PFS curve of Kang Ai injection group on the economic conclusion.

**Results:**

The base-case result indicated that Kang Ai injection group provided 0.217 incremental quality-adjusted life years (QALYs) at an incremental cost of $103.38 compared with Shenqi Fuzheng injection group. The ICER was $476.41/QALY, which was much lower than the willingness to pay threshold of one time the GDP per capita of China in 2022 ($12,070/QALY). Deterministic sensitivity analysis result showed that ICER was most sensitive to the changes in odds ratio (OR) value. The probabilistic sensitivity analysis confirmed the robustness of base-case analysis results. The scenario analysis result showed that by using Log-Normal distribution to fit the PFS curve of Kang Ai injection group and shortening the time horizon to 5 years, the ICER was $4,081.83/QALY, which was still much lower than the willingness to pay threshold.

**Conclusion:**

Kang Ai injection combined with platinum-based chemotherapy appeared to be more cost-effective for the treatment of advanced NSCLC than Shenqi Fuzheng injection combined with platinum-based chemotherapy.

## 1 Introduction

Lung cancer is one of the most common cancer types and can be classified as non-small cell lung cancer (NSCLC) and small cell lung cancer (SCLC) (1). Of overall lung cancer cases, NSCLC accounts for up to approximately 85% and can be classified as adenocarcinoma, squamous cell carcinoma, and large cell carcinoma (2, 3). As statistics from Globocan 2020, the number of new cases of lung cancer reached 2,206,771, accounting for 11.4% of all new cancer cases, and the number of new deaths of lung cancer reached 1,796,144, accounting for 18% of all cancers in 2020 (4).

China is also one of the countries with a high incidence rate of lung Cancer. According to the latest statistics published by National Cancer Center, the incidence rate of lung cancer ranks first among all cancers, about 59.89/100,000 (5). Although the survival rate of all cancers has been improved in recent years, the survival rate of lung cancer is still relatively low. From 2012 to 2015, the survival rate of lung cancer in men was 16.8%, which was 62.5% different from the highest survival rate of thyroid cancer, and the survival rate of women was 25.1%, which was also very low (6). When diagnosed as NSCLC, more than one-third of patients are locally advanced and have lost the best opportunity for surgical treatment (7). According to the guideline, platinum-based two-drug regimen is the standard chemotherapy regimen for the first-line treatment of advanced NSCLC (8). However, chemotherapy often causes adverse reactions, such as anemia, neutropenia, thrombocytopenia, fatigue, and poor appetite, which reduce patient immunity and seriously affect quality of life of patients. Hence, in east Asia, particularly in China, Chinese patent medicines combined with chemotherapy are used in the treatment of advanced NSCLC, in order to reduce the side effects of chemotherapy and improve clinical efficacy (9).

Currently, there are several Chinese patent medicines available for the treatment of NSCLC, including Kang Ai injection, Shenqi Fuzheng injection, Kanglaite injection, and Aidi injection. Among them, Kang Ai injection is primarily made from ginseng and astragalus as the main raw materials. Under the guidance of the basic theory of traditional Chinese medicine, the purpose of treating cancer patients can be achieved by removing toxins and improving immunity. Several meta-analysis studies have confirmed that Kang Ai injection combined with platinum-based chemotherapy was more beneficial to patients with advanced NSCLC when compared to chemotherapy alone, which can be more effective in improving the clinical efficacy, reducing chemotherapy toxicity, decreasing the incidence rate of adverse reactions and regulating the tumor immune function (10–12).

So far, the clinical application of Kang Ai injection in the treatment of NSCLC patients has been recognized, but the cost-effectiveness of Kang Ai injection is still unclear. Therefore, this study conducted an economic evaluation for the cost-effectiveness of Kang Ai injection in China to better inform clinical decision-making and provide cost-effectiveness evidence for the reimbursement policy. At present, almost all clinical trials of Chinese patent medicines combined with chemotherapy for the treatment of cancer patients have not reported Kaplan–Meier curves, and there are few normative economic evaluation researches with high quality on Chinese patent medicines (13). Hence, the other purpose of this study is to estimate survival curves based on published literature and validated methods, which may provide reference for future economic evaluation research about Chinese patent medicines (14, 15).

## 2 Materials and methods

### 2.1 Target population

The target population of the model was based on the clinical trial of Kang Ai injection. This trial included 326 patients from multiple hospitals in China, all aged 18–75 years and diagnosed with IIIB–IV stage NSCLC according to pathology or cytology (16). The initial average age of patients was assumed to be 59 years old in our research. To calculate costs, we also assumed that average body surface area was 1.6 m^2^ and creatinine clearance rate was 80 ml/min.

### 2.2 Interventions and comparators

At present, there are a variety of Chinese patent medicines for adjuvant treatment in clinical practice, among which Shenqi Fuzheng injection can be combined with chemotherapy for adjuvant treatment of NSCLC, and it is also in the National Reimbursement Drug List (NRDL). Therefore, Shenqi Fuzheng injection combined with chemotherapy was selected as the control group in this study. The intervention of treatment group is Kang Ai injection combined with gemcitabine or paclitaxel plus platinum-based chemotherapy. All drugs were administered with a cycle of 3 weeks.

#### 2.2.1 Treatment group

The dosage of Kang Ai injection was 60 ml and intravenous infusion was administered from day 1 to day 14 of each cycle. The dosage of gemcitabine was 1,250 mg/m^2^ and intravenous infusion was administered on day 1 and day 8 of each cycle. The dosage of paclitaxel was 150 mg/m^2^ and intravenous infusion was administered on the first day of each cycle. The dosage of cisplatin was 75 mg/m^2^ and intravenous infusion was administered on the first day of each cycle. The dosage of carboplatin was 5 mg/ml per minute in the area under the curve (AUC) and intravenous infusion was administered on the first day of each cycle.

#### 2.2.2 Control group

The dosage of Shenqi Fuzheng injection was 250 ml and intravenous infusion was administered from day 1 to day 14 of each cycle. The usage and dosage of gemcitabine or paclitaxel plus platinum-based chemotherapy were consistent with Kang Ai group.

### 2.3 Model design

The Markov model is applicable for the simulation of disease progression among a finite number of health states. Under appropriate assumptions, it is convenient to simulate patients’ long-term health state changes. Many published studies built a three-state Markov model to evaluate the cost-effectiveness of drugs for patients with NSCLC (17–20). This demonstrated the suitability of the Markov model. Therefore, we chose the Markov model for simulation.

**FIGURE 1:**
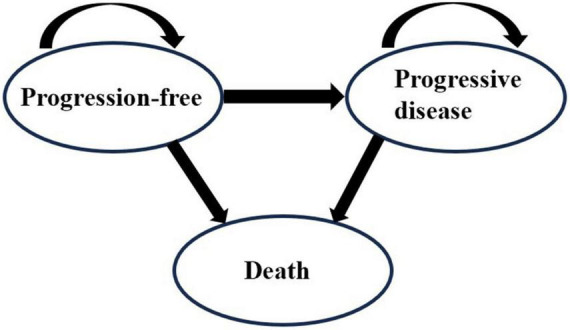
Model structure of the Markov model.

A Markov cohort simulation model was developed with Microsoft Excel (Version 2019) to estimate the costs and outcomes of Kang Ai injection or Shenqi Fuzheng injection combined with platinum-based chemotherapy for the treatment of patients with advanced NSCLC. The Markov model incorporated three states: progression-free (PF), progressive disease (PD) and death, as shown in Figure 1. We assumed that all patients were in the PF state when they entered the model at the very beginning. According to the drug regimens, the cycle length was set as 3 weeks and the time horizon was 10 years based on the poor prognosis of advanced NSCLC and follow-up period, allowing nearly 100% of the patients in all treatment groups to reach the state of death. The main outcomes of the model were total costs, life years, quality-adjusted life years (QALYs), and incremental cost-effectiveness ratio (ICER).

### 2.4 Survival analysis

The Kaplan–Meier curves for progression-free survival (PFS) and overall survival (OS) of Shenqi Fuzheng injection combined with chemotherapy in the first-line treatment of advanced NSCLC have not been reported in the existing clinical trials. Therefore, we could not recreate pseudo-individual-level data to fit the survival curves. Meanwhile, although individual patient data from clinical trials of Kang Ai injection can be obtained, the OS curve is not mature and only the PFS curve of Kang Ai injection can be analyzed for survival. Because of the above limitations, this study firstly extrapolated the objective response rate (ORR) value of Shenqi Fuzheng injection group based on the results of reported ORR value of Kang Ai group and existing network meta-analysis (NMA). According to the existing research, the median PFS (mPFS) and median OS (mOS) were calculated by ORR values. Then survival curves were estimated by the method of declining exponential approximation of life expectancy (DEALE), which is based on the assumption that survival follows a declining exponential function (14, 15).

The ORR value of Kang Ai injection combined with platinum-based chemotherapy in first-line treatment of advanced NSCLC was 28.2% (16). Li et al. (21) conducted a NMA on clinical trials of 10 Chinese traditional medicine injections combined with gemcitabine plus platinum-based chemotherapy in the treatment of advanced NSCLC, and found that the odds ratio (OR) value of ORR of Kang Ai injection group compared with Shenqi Fuzheng injection group was 1.23. Then, we calculated that the ORR value of Shenqi Fuzheng injection group was 24.2%. Rui et al. (22) reviewed and analyzed clinical trials of drugs in the treatment of advanced NSCLC in China, and obtained the correlation between clinical short-term surrogate endpoint indicators (ORR, etc.) and endpoint indicators (mPFS and mOS). The calculation equations are as follows:


l⁢n⁢(m⁢P⁢F⁢S)=2.36×O⁢R⁢R+0.84

m⁢O⁢S=32.98×O⁢R⁢R+4.96

Ln(mPFS) refer to natural logarithm of mPFS; mPFS refer to median progression-free survival; mOS refer to median overall survival; ORR refer to objective response rate.

According to the above equations, the mPFS, mOS of Shenqi Fuzheng injection group and mOS of Kang Ai injection group were calculated. The results are shown in Table 1. After obtaining the mPFS, mOS of Shenqi Fuzheng injection group and mOS of Kang Ai injection group, the three curves were fitted with exponential distribution. In order to eliminate the influence of different distribution selection, the PFS curve of Kang Ai injection group was also fitted with exponential distribution. The related parameters of survival curves are shown in Table 2. Transition probabilities were calculated from the estimated survival curves. Age- and sex-specified general population mortality which derived from the sixth national census results were extracted to obtained probability of PF to PD state (23).

**TABLE 1:** Objective response rate and median PFS/OS parameters.

	Kang Ai injection group	Shenqi Fuzheng injection group
ORR	28.2%	24.2%
mPFS (months)	6.17*	4.09
mOS (months)	14.26	12.94

ORR, objective response rate; mPFS, median progression-free survival; mOS, median overall survival.

*Obtained from the clinical trial of Kang Ai injection.

**TABLE 2:** Parameters of survival curves.

Group	Survival curves	Distribution	Parameters
Kang Ai injection group	PFS	Exponential	λ = 0.06, 431
	OS	Exponential	λ = 0.03, 316
Shenqi Fuzheng injection group	PFS	Exponential	λ = 0.11, 175
	OS	Exponential	λ = 0.03, 680

PFS, progression-free survival; OS, overall survival.

### 2.5 Resource utilization and costs

From the perspective of Chinese healthcare system, we only considered direct medical costs, which included drug acquisition cost, follow-up cost, disease management cost, subsequent treatment cost, and end life cost. Due to the synergistic and attenuating effects of Chinese patent medicines in NSCLC patients, the incidence rate of grade 3 or 4 adverse events (AEs) is lower than 5% (16, 24). Therefore, the costs associated with AEs were not considered in this study. All the costs were updated to 2023 US dollars ($1 = ¥7.10) (25).

Drug acquisition cost included the costs of Kang Ai injection, Shenqi Fuzheng injection, platinum (carboplatin or cisplatin) and gemcitabine or paclitaxel. The costs of Kang Ai injection, Shenqi Fuzheng injection were sourced from current price provided from the original manufacturer. Costs of carboplatin and cisplatin were the median bidding price gathered across provinces in recent 1 year. The costs of gemcitabine and paclitaxel were sourced from median price in the fifth volume-based procurement scheme. In the calculation of drug acquisition cost, the ratio of patients to gemcitabine or paclitaxel was assumed to be 1:1, and the ratio of patients to carboplatin or cisplatin was assumed to be 1:1.

In the first cycle of PF health state, the follow-up cost included the costs of routine blood test, routine urine test, and blood chemistry examination. In the second cycle of PF health state, the follow-up cost included the costs of computed tomography (CT), magnetic resonance imaging (MRI), B-ultrasound, chest X-ray, bone scan, routine blood test, routine urine test, and blood chemistry examination. In the rest cycles of PF health state, the follow-up cost included the costs of CT, MRI, B-ultrasound, chest X-ray, and bone scan. The follow-up cost of each PD health state cycle included CT, MRI, B-ultrasound, chest X-ray, and bone scan.

The cost of disease management was calculated according to PF and PD health state, respectively. The disease management cost of the first two cycles of PF health state included costs of diagnosis, intravenous injection, bed, and nursing. The disease management cost of the remaining cycles of PF health state and PD health state included costs of diagnosis, bed, and nursing.

Follow-up cost and disease management cost were derived from the medical service price lists of 13 provinces including Beijing, Shanghai, Hebei, Shanxi, Jiangsu, Zhejiang, Chongqing, Fujian, Shanxi, Shandong, Guangzhou, Henan, and Jiangxi. Subsequent treatment cost and end life cost were derived from the literature (26, 27). Key inputs related to costs are listed in Table 3.

**TABLE 3:** Summary of model inputs and ranges.

Model inputs	Deterministic	Minimum	Maximum	Distribution	Source
**Drug acquisition cost/$**					
Kang Ai injection (20 mg)	4.77	3.82	4.77	Constant	Current price provided from the original manufacturer
Shenqi Fuzheng injection (250 ml)	15.94	12.76	15.94	Constant	Current price provided from the original manufacturer
Gemcitabine (200 mg)	8.44	1.13	9.01	Gamma	Median price in the fifth volume-based procurement scheme, 2021
Paclitaxel (100 mg)	23.80	19.04	28.56	Constant	Median price in the fifth volume-based procurement scheme, 2021
Paclitaxel (30 mg)	9.99	9.58	10.39	Gamma	Median price in the fifth volume-based procurement scheme, 2021
Cisplatin (20 mg)	1.96	1.03	2.44	Gamma	The median bidding price gathered across provinces in recent 1 year
Carboplatin (50 mg)	11.13	4.23	13.80	Gamma	The median bidding price gathered across provinces in recent 1 year
Carboplatin (100 mg)	21.73	3.73	23.66	Gamma	The median bidding price gathered across provinces in recent 1 year
Proportion of cisplatin	0.5	0	1	Beta	Assumption
Proportion of GP	0.5	0	1	Beta	Assumption
**Follow-up cost per time/$**					
CT	15.21	8.45	39.44	Gamma	Median value collected from the medical service price lists of 13 provinces
MRI	49.30	33.24	59.15	Gamma	Median value collected from the medical service price lists of 13 provinces
B-ultrasound	4.23	2.11	7.61	Gamma	Median value collected from the medical service price lists of 13 provinces
Chest X-ray	0.70	0.56	1.13	Gamma	Median value collected from the medical service price lists of 13 provinces
Bone scan	42.25	28.17	63.38	Gamma	Median value collected from the medical service price lists of 13 provinces
Routine blood test	1.20	0.35	3.90	Gamma	Median value collected from the medical service price lists of 13 provinces
Routine urine test	0.56	0.14	2.54	Gamma	Median value collected from the medical service price lists of 13 provinces
Blood chemistry examination	25.51	17.04	52.32	Gamma	Median value collected from the medical service price lists of 13 provinces
**Disease management cost per time/$**					
Diagnosis	3.17	1.55	9.86	Gamma	Median value collected from the medical service price lists of 13 provinces
Intravenous injection	1.13	0.85	1.69	Gamma	Median value collected from the medical service price lists of 13 provinces
Bed	5.63	1.90	9.15	Gamma	Median value collected from the medical service price lists of 13 provinces
Nursing	2.82	0.85	4.23	Gamma	Median value collected from the medical service price lists of 13 provinces
Subsequent treatment cost/ –Y	2,028.0	11,622.4	2,433.6	Gamma	(26)
End life cost/ –Y	2,176.0	1,740.8	2,611.2	Gamma	(27)
**Health utility**					
PF health state	0.804	0.643	0.965	Beta	(28)
PD health state	0.321	0.257	0.385	Beta	(28)
**Clinical parameter**					
OR	1.23	0.79	1.92	Lognormal	(21)
Discount rate	0.05	0	0.08	Constant	(30)

### 2.6 Utility inputs

The health utilities were derived from the research of Nafees et al. (28), which was based on the original Nafees et al. (29) study published in year 2008. Because of the limitations of methodology and geographical applicability in the original study, the study published in 2016 used time trade-off (TTO) method to capture utilities for metastatic NSCLC from local populations in the United Kingdom, Australia, France, China, Taiwan, and Korea. From the research, we applied the PF utility of 0.804 and PD utility of 0.321 based on the results of Chinese people. The details of utility parameters are displayed in Table 3.

According to the China Guidelines for Pharmacoeconomics Evaluations (2020), the annual discount rate applied to costs and health outcomes was 5% (30).

### 2.7 Sensitivity analysis and scenario analysis

We carried out one-way sensitivity analysis, probabilistic sensitivity analysis, and scenario analysis to evaluate the robustness of base-case results. According to the guidelines, the willingness-to-pay (WTP) threshold value for QALY is recommended to be 1–3 times of the GDP per capita (30). We deemed one time the GDP per capita of China in 2022 ($12,070/QALY) as the threshold.

For one-way sensitivity analysis, we varied a number of parameters and displayed 10 most influential parameters in tornado diagrams. For parameters, of which we could not obtain the upper and lower limits, we assumed a 20% fluctuation from the baseline value. For drugs included in the NRDL, such as Kang Ai injection and Shenqi Fuzheng injection, the prices of these drugs were stable. Hence, we assumed that the upper limits of the drug prices were the prices used in base-case analysis and the lower limits of the prices were taken as 80% of the base-case prices. Besides, we conducted a probabilistic sensitivity analysis (PSA) via Monte Carlo simulation in which every key input was assumed to fit a theoretical distribution. The results of 1,000 iterations were drawn in the form of scatter plot and cost-effectiveness acceptability curves (CEACs). Parametric distributions and ranges used in the analysis are displayed in Table 3.

In order to further explore the uncertainty of the economic results, we carried out a scenario analysis. In order to eliminate the impact of different distributions, the PFS curve of Kang Ai injection group was fitted with an exponential distribution in the base-case analysis. While in the scenario analysis, the PFS curve of Kang Ai group was fitted with a Log-Normal distribution, which was the best-fitting distribution. The time horizon was also reduced to 5 years.

## 3 Results

### 3.1 Base-case results

In the 10 years of simulation, per patient in Kang Ai injection group obtained 0.914 QALYs and 1.602 life years and needed to pay $5,997.32. In Shenqi Fuzheng injection group, per patient obtained 0.697 QALYs and 1.459 life years and needed to pay $5,893.94. The ICER derived from Kang Ai injection group compared with Shenqi Fuzheng injection group was $476.41/QALY, which was less than the willingness to pay threshold of one time the GDP per capita of China in 2022. Hence, Kang Ai injection demonstrated a supremely good cost-effectiveness in the base-case analysis. The costs, life years gained, QALYs and ICERs in base-case analysis are given in Table 4.

**TABLE 4:** Base-case analysis results.

	Shenqi Fuzheng injection group	Kang Ai injection group	Increment
Total cost ($)	5,893.94	5,997.32	103.38
Kang Ai injection costs		362.79	
Shenqi Fuzheng injection costs	376.48		
Chemotherapy costs	311.30	333.89	
Follow-up costs	790.58	868.16	
Disease management costs	363.30	398.07	
Subsequent treatment costs	2,037.93	2,037.93	
End life costs	2,014.35	1,996.48	
Life years	1.459	1.602	0.143
QALYs	0.697	0.914	0.217
ICER			476.41

All results are discounted. QALYs, quality-adjusted life years; ICER, incremental cost-effectiveness ratio.

### 3.2 Deterministic sensitivity analysis

**FIGURE 2:**
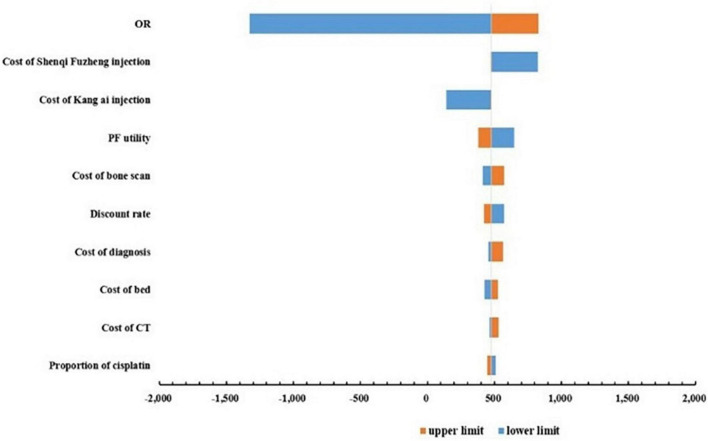
Tornado diagram of sensitivity analysis.

One-way sensitivity analyses were conducted to test the robustness of the base-case ICER, and the results were shown in the form of tornado diagrams in Figure 2. The base-case ICER was most sensitive to changes in OR value, followed by the cost of Shenqi Fuzheng injection, cost of Kang Ai injection, the PF health state utility. The remaining parameters had less influence on the base-case results. It is worth noting that changes in all parameters cannot reverse the base-case results.

### 3.3 Probabilistic sensitivity analysis

**FIGURE 3:**
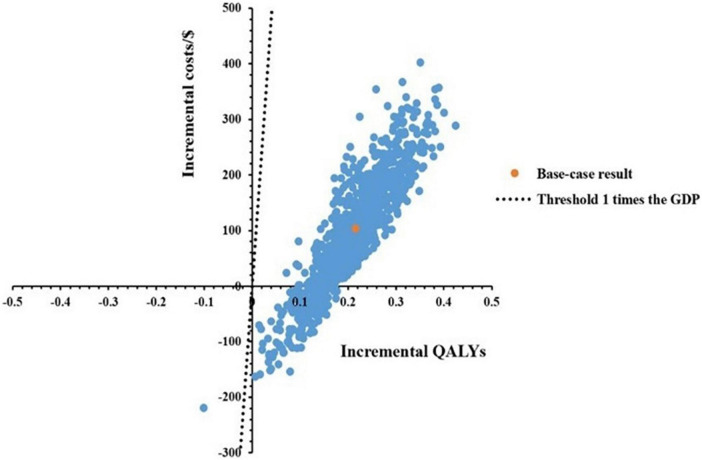
Cost-effectiveness scatter plot results with 10,000 iterations.

A total of 1,000 Monte Carlo simulations were conducted to obtain a scatter plot, as shown in Figure 3. Under the threshold of one time the GDP per capita, the scatters which represented the ICERs of Kang Ai injection were almost all below the threshold line, meaning the probability for Kang Ai injection to be cost-effective when compared with Shenqi Fuzheng injection was 100%.

**FIGURE 4:**
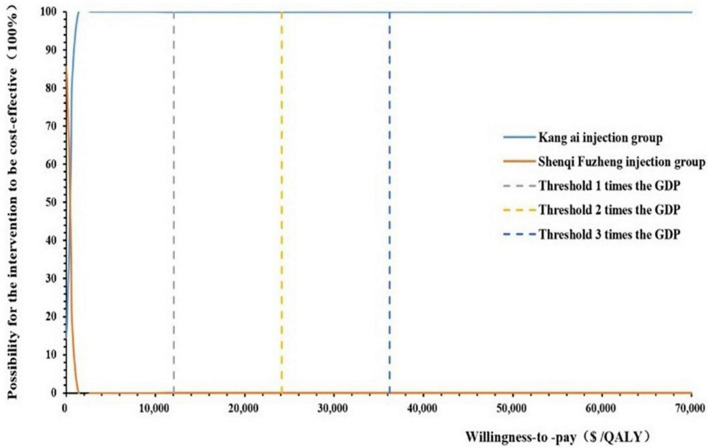
Cost-effectiveness acceptability curves.

When willingness to pay threshold varied from $0 to $70,000 per QALY gained, we drawn a cost-effectiveness acceptability curve (CEAC), as shown in Figure 4. According to the CEAC curve, Kang Ai injection group had a nearly 100% probability to be cost-effective under the threshold of 1–3 times GDP per capita.

### 3.4 Scenario analysis results

We considered seven parametric distributions to fit the PFS curve of Kang Ai group: exponential, gamma, generalized F, generalized gamma, weibull, loglogistic, and log-normal. The evaluation index of assessment of fit was based on Akaike information criterion, Bayesian information criterion, as presented in Table 5. SurvHE package was used to fit various survival models in R (version of 4.2.1). Information about individual patient data of Kang Ai injection are presented in Supplementary Table 1.

**TABLE 5:** Results of fitting to the observed data.

		Exponential	Gamma	Generalized F	Generalized gamma	Weibull	Loglogistic	Log-normal
Kang Ai injection PFS	AIC	266.09	235.81	234.27	234.28	240.57	235.45	232.41
	BIC	272.19	244.96	249.52	246.47	249.72	244.60	241.56

Finally, the Log-Normal distribution was used, which was the best-fitting distribution, to fit the PFS curve of Kang Ai group. The value of natural logarithm of the mean and standard deviation parameters were 1.91 and 1.66, respectively. With a time horizon of 5 years, the ICER significantly increased from $476.41/QALY in the base-case analysis to $4,081.83/QALY, but still far below the willingness-to-pay threshold of one time the GDP per capita of China in 2022 ($12,070/QALY), indicating that Kang Ai injection group was to be cost-effective when compared with Shenqi Fuzheng group. Scenario analysis results are displayed in Table 6.

**TABLE 6:** Scenario analysis results.

Regimen	Costs	Life years	QALYs	ICER
Kang Ai injection group	5,951.06	1.574	0.720	4,081.83
Shenqi Fuzheng injection group	5,798.08	1.415	0.683	

## 4 Discussion

Traditional Chinese medicine has been developed in China for thousands of years, which is a treasure of the Chinese nation. In recent years, China has issued a series of policy papers to support the development of traditional Chinese medicines (31, 32). In the process of entering the NRDL, China focuses on supporting Chinese medicines and ethnic medicines adhering to the principle of “attaching equal importance to Chinese and Western medicines.” Therefore, the number of Chinese patent medicines in the NRDL has increased year by year and the economic evaluation of these medicines plays a very important role during the process. This is the first study to investigate the cost-effectiveness of Kang Ai injection combined with platinum-based chemotherapy as the first-line treatment for advanced NSCLC patients in China. Our findings serve to facilitate healthcare resource allocation and provide cost-effectiveness evidence for decision-making. Meanwhile, our research designs aim to provide reference for future economic evaluation research about Chinese patent medicines in neoplasms.

Because of the lack of clinical trial data, published and innovative methods were used to generate the survival curves Based on the results of reported ORR and OR values, we extrapolated the mPFS, mOS, and then survival curves were estimated based on the assumption that survival follows a declining exponential function. We built a Markov model with a time horizon of 10 years. Transition probabilities were calculated from the estimated survival curves and age- and sex-specified general population mortality. The principal finding of base-case analysis was that the ICER of Kang Ai injection group had an overwhelming advantage over Shenqi Fuzheng injection group, which was much lower than the one-time GDP per capita, indicating the excellent cost-effectiveness. One-way sensitivity analysis indicated that OR value imposed considerable uncertainty on the base-case ICER. The probabilistic sensitivity analysis confirmed the robustness of base-case analysis results. Under the willingness to pay threshold of 1–3 times GDP per capita, Kang Ai injection group had a nearly 100% probability to be cost-effective. Scenario analysis found that although best-fitting distribution for PFS curve of Kang Ai injection group multiplied the value of ICER, the ICER was still much lower than the willingness to pay threshold.

In recent years, researches of cost effectiveness analysis on Chinese patent medicines in neoplasm have made some progress in both quantity and quality, but there are still many deficiencies. The main reasons are the lack of clinical trial data of Chinese patent medicines with high quality, and the selection of research design, time horizon and evaluation methods cannot fully reflect the advantages of Chinese patent medicines (33). Among the published economic evaluation studies of Chinese patent medicines in neoplasms, the longest time horizon reported was 1 year. Most of these studies were published several years ago and may not accurately reflect the current costs and cost-effectiveness of these drugs (34). Short time horizon is hard to capture all the economic costs and health outcomes of Chinese patent medicines. Only one research adopted QALYs as an indicator of health outcomes (35). Wang et al. (36) systematically evaluated the quality of published pharmacoeconomics studies on Chinese patent medicines for neoplasms and indicated that most studies were not model-based and only effective rate was used as the health outcome, and less than half of them conducted uncertainty analysis. In our study, we actively avoided these deficiencies and carried out a complete and standardized economic evaluation of Chinese patient medicines.

To be noted, our research has some limitations which may generate bias or uncertainty to the explanation of study outcomes. To begin with, this study assumed that Kang Ai injection and Shenqi Fuzheng injection were combined with gemcitabine or paclitaxel plus platinum-based chemotherapy, and the effect of combination with other platinum-based chemotherapy on the results was not considered. Also, due to the lack of data, this study assumed that an equal proportion of patients receive gemcitabine plus platinum-based chemotherapy and paclitaxel plus platinum-based chemotherapy. The proportion of patients receiving cisplatin and carboplatin was also assumed to be the same. Secondly, because of the low incidence rate of AEs, this study did not consider the occurrence of AEs and may not be able to fully and accurately present the true costs and health outcomes of the two treatment groups. Thirdly, mPFS and mOS were obtained by ORR, which could generate uncertainty. Additionally, we had to rely on the assumption of declining exponential function for survival curves to calculating transition probability because no clinical trial reported OS and PFS curves of Shenqi Fuzheng injection.

## 5 Conclusion

This economic evaluation revealed that Kang Ai injection combined with platinum-based chemotherapy appeared to be cost-effective for patients diagnosed with advanced NSCLC in China when compared with Shenqi Fuzheng injection combined with platinum-based chemotherapy.

## Data availability statement

The original contributions presented in this study are included in the article/Supplementary material, further inquiries can be directed to the corresponding author.

## Author contributions

JZ: Data curation, Formal analysis, Methodology, Software, Writing – original draft. LT: Validation, Writing – review & editing.

## Supplementary material

Supplementary Table 1 Individual patient data of Kang Ai injection.
